# Correspondence

**Published:** 1890-09

**Authors:** 


### Correspondence.

NEW YORK, July 30, 1890.

During the course of a conversation with the writer the other day, one of the best known and most successful gynecologists of this city expressed himself in the following manner: The operation of laparotomy for the removal of diseased conditions within the peritoneum is not resorted to now, with quite as much frequency as in former times. Within the past year or so, laparotomists are beginning to realize the unfortunate consequences which frequently follow this operation, and particularly when the condition for which it was performed, was not properly understood. The tide now seems to have set in in the opposite direction and it is quite evident to the mind of any reasoning physician that this change of base had to come. Within the past year or so careful and conservative gynecologists have begun to doubt whether the mere removal of diseased conditions within the abdominal cavity does really effect a permanent and absolute cure. It is no unusual thing for patients who have had a laparotomy performed on them to apply again for treatment six months after the operation with the same train of symptoms for the relief of which the laparotomy was undertaken.

The ultimate results of the removal of diseased conditions within the abdominal cavity seem to have been overlooked by many operators who have published their reports as cures as soon as the patient had recovered from the immediate effects of the operation, leaving the subsequent history to be taken for granted.

It makes no difference how the operation is performed, with what skill, or care, or what the condition of the patient may be, even though she may promptly recover from the effects of the operative procedures, still time and experience have proved that adhesions very frequently follow from the fibrogenetic exudate thrown out during the period of the inflammatory changes taking place within the abdominal cavity that bind together the floating viscerea, and in that way interfere with the circulation and consequent nutrition of the parts.

Furthermore, in order to perform a laparotomy with any degree of satisfaction or success, a large incision must be made in the abdomen which heals by cicatrization, and cicatricial tissue, as every one knows, in time undergoes a weakening, thinning and stretching that is frequently followed by a hernial protrusion.

This question of laparotomy has, within the last few years, been discussed in a general and systematic way by very many of our ablest gynecologists, and they have reached the conclusion that success immediately following an operation of this sort must not be regarded as synonymous with a cure of the condition for which it was undertaken.

As an evidence that this reaction is not of an ephemeral character and that it has not been brought about by enemies to all substantial progress, is proven by the fact that even so great and successful an operator as Keith, of London, to-day claims that the majority of pathological formations within the peritoneum are amenable to treatment by agents which do not entail mutilation of the abdominal parieties by abdominal section. Electricity has been taken up and its praises loudly vaunted, and there are not a few who claim for it results in the treatment of inflammatory, neuralgic and vascular conditions of the serous lining of the abdomen, the uterus and the uterine appendages, which surgeons are unable to obtain when applying the same agent to other parts of the body. For instance, one author says that he will reduce a voluminous fibroid with as much ease and rapidity under action of electricity as the sun will melt away a snow bank; that this new panacea will arrest congestion, subdue inflammation and promote tissue metamorphosis with marvelous rapidity in every variety of tumor, cancer not excepted, that may affect the female genitals.

From the very fact that so much is claimed with such force and earnestness, and no doubt with more or less truth, for electricity in the treatment of pathological conditions within the peritoneal cavity, it may be safe to conclude that these morbid conditions can be dealt with very often successfully by constitutional agencies alone, and that treatment applied both internally and locally will, in the majority of instances, succeed not only in completly arresting pathological changes, but in permanently curing the diseased condition as well.

As an illustration of the laxity with which the laws are administered in this city, may be cited the case of Dr. McGonigal, that veteran abortionist, who is now figuring in the courts of New York because he has plyed his old "trade" once too often. This old offender has all his lifetime travelled along the thorny and dangerous path of unrighteousness, and has been indicted on more than one occasion for murder. As a matter of fact, there was an indictment hanging over him at the very time he committed this last offence, which brought him within the power of the law. Public opinion is now so much aroused on this subject that it would not be any longer safe for any court or jury to interfere with the course of justice, and it is generally believed that McGonigal will spend the remainder of his days at least behind prison bars. His career has been a bad one, its termination, as regulated by the courts, will be deplored by but few.

The reputable physicians of New York will not shed many tears over the issue, whatever it may be, and the profession of medicine will be saved from the reproach of having such an unworthy member in its ranks, a man who falsified by his life the principles he was supposed to live up to, and whose mission has been to destroy not to save life. Never since the days of Mme Restell, another noted abortionist of this city, has the public mind been so thoroughly aroused.

The inventive genius of Dr. Wyeth, has again been at work in devising a method for performing bloodless amputation at the hip joint. Profs. Fluhrer and McBurney have both tried it and have found it satisfactory in every respect. The indications now are that operations about the hip joint, as a result of this new method of procedure, will be as free from danger in the future as those done in any other part of the body.

Among the recent matriculants at the New York Polyclinic were G. Hashimato, M. D., of Kob, Japan, and Dzan Tse Zeh, of Soochow, China. The latter, who has taken the English name of C. K. Marshall, is also a minister of the gospel, but intends to devote his life henceforth to the caring for the bodily ills of the 60,000 or more Mongolians of New York, Brooklyn and Jersey City. With this large field to work in, it can be readily understood that in the domain of medicine, Dzan Tse Zeh, M. D., will enjoy a very lucrative practice.

The discovery of a genuine leper in a boarding house in this city has excited no small amount of uneasiness in certain quarters. The patient, who is a native of Yucatan, has resided in this country for some time and it is difficult to understand how he managed to acquire this disease. He has not been made aware of the terrible nature of the malady as yet, but is kept isolated in one of the city hospitals, awaiting the arrival of his parents. There seems to be no possible mistake in the diagnosis that has been made in this young man's case.

There is a deep stagnation in medical matters at present, and there is little or nothing of any moment occurring in the hospitals. We are now in the midst of the regular summer season, and all who are able at all to do so, have left the city for the seaside or the mountains. Most of the lights of the medical world have sped across the water and life in New York is at present not worth living for.

The New York Polyclinic has had an unusually successful year of it and indications all point to a very large attendance during the fall and winter season.

